# xGNN4MI: explainability of graph neural networks in 12-lead electrocardiography for cardiovascular disease classification

**DOI:** 10.1038/s41746-026-02367-1

**Published:** 2026-02-06

**Authors:** Miriam Cindy Maurer, Philip Hempel, Kristin Elisabeth Steinhaus, Hryhorii Chereda, Marcus Vollmer, Dagmar Krefting, Nicolai Spicher, Anne-Christin Hauschild

**Affiliations:** 1 Department of Medical Informatics, University Medical Center Göttingen, Göttingen, Germany; 2 Campus Institute Data Science (CIDAS), University of Göttingen, Göttingen, Germany; 3 Clinic of Cardiology and Pneumology, University Medical Center Göttingen, Göttingen, Germany; 4 Biomedical Network Science Lab, Department of Artificial Intelligence in Biomedical Engineering, Friedrich-Alexander University Erlangen-Nürnberg, Erlangen, Germany; 5 Institute of Bioinformatics, University Medicine Greifswald, Greifswald, Germany; 6 German Centre for Cardiovascular Research (DZHK), Partner Site Greifswald, Greifswald, Germany; 7 Department of Health Technology, Technical University of Denmark, Copenhagen, Denmark; 8 Institute for Predictive Deep Learning in Medicine and Healthcare, Justus-Liebig University Gießen, Gießen, Germany

**Keywords:** Cardiology, Computational biology and bioinformatics

## Abstract

The clinical deployment of artificial intelligence (AI) solutions for assessing cardiovascular disease (CVD) risk in 12-lead electrocardiography (ECG) is hindered by limitations in interpretability and explainability. To address this, we present xGNN4MI, an open-source framework for graph neural networks (GNNs) in ECG modeling for interpretable CVD prediction. Our framework facilitates modeling clinically relevant spatial relationships between ECG leads and their temporal dynamics. We integrated explainable AI (XAI) and developed a task-specific XAI evaluation and visualization workflow to identify ECG leads crucial to the model’s decision-making process, enabling a systematic comparison with established clinical knowledge. We evaluated xGNN4MI on two challenging tasks: diagnostic superclass classification and localization of myocardial infarction. Our findings show that the interpretable ECG-GNN models demonstrate good performance across the tasks. XAI analysis revealed clinically meaningful training effects, such as differentiating between anteroseptal and inferior myocardial infarction. Our work demonstrates the potential of ECG-GNNs for providing trustworthy and interpretable AI-based CVD diagnosis.

## Introduction

Cardiovascular diseases (CVDs) are one of the major global health challenges, contributing to a significant proportion of morbidity and mortality worldwide^1^. Early and accurate detection of CVDs is crucial for timely intervention and effective patient treatment. Electrocardiography (ECG) is the standard method for a quick assessment of the heart due to its affordability and low risk, as it is a non-invasive procedure. However, the complexity of CVD, combined with the variability in ECG patterns, often poses a challenge for physicians in ECG interpretation. Although ECG devices output certain values and disease indications, the final diagnosis remains highly dependent on the physician’s training, certifications, experience, and knowledge^2^. Unfortunately, physicians’ competency is often lacking in resource-limited settings, particularly in the global south^3^.

Myocardial infarction (MI) is a critical condition that is characterized by the occurrence of irreversible myocardial cell death, typically resulting from prolonged ischemia due to obstruction of the coronary arteries, leading to a reduction of blood flow^4^. According to the World Health Organization (WHO), more than 15.2 million fatalities per year are attributable to MI alone^1^. Therefore, the timely detection and accurate localization of MI are essential for initiating appropriate therapeutic interventions, which can significantly reduce mortality and improve long-term outcomes.

**Fig. 1:**
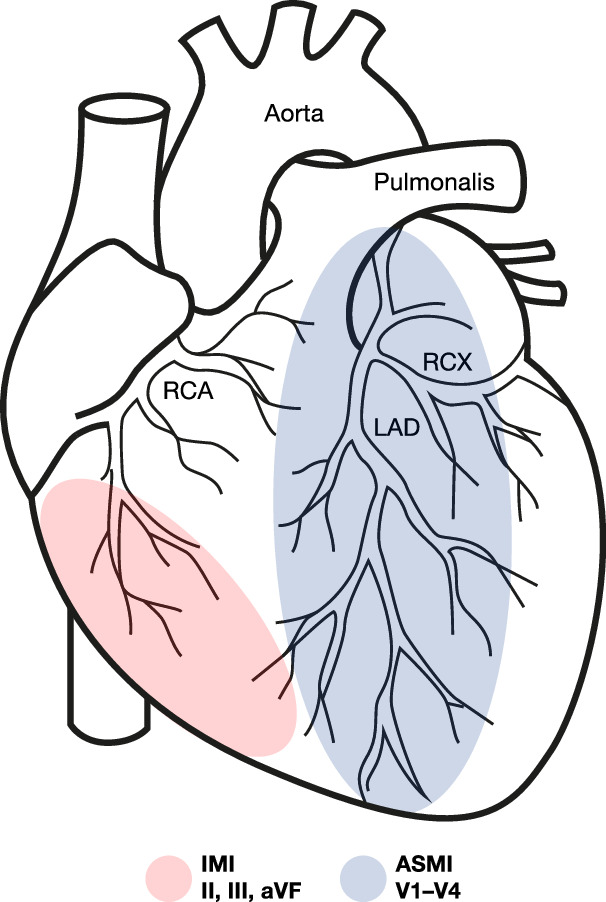
Clinical association between ECG leads and MI locations. Leads II, III, and aVF (inferior leads) are used to detect IMI. Leads V1–V4 (precordial leads) are essential for detecting ASMI. Adapted from cardiology textbook knowledge.

Changes in specific ECG leads are indicative of different regions of MI, as illustrated in Fig. 1. Inferior myocardial infarction (IMI) is typically indicated by ST-elevations in leads II, III, and aVF, while anteroseptal myocardial infarction (ASMI) is detected through leads V1–V4. These associations stem from the anatomical relationship between lead placements and the vascular territories supplied by coronary arteries, such as the right coronary artery (RCA) and the left anterior descending artery (LAD). Accurate ECG-based identification of the infarcted region is imperative for effective clinical decision-making, as well as enhancing the efficacy of reperfusion strategies and post-infarction management^5,6^.

In recent years, deep learning (DL) has emerged as a powerful alternative approach for ECG-based diagnosis and risk assessment, with numerous studies demonstrating its effectiveness^7–9^. Despite their success, these end-to-end models are often criticized for their lack of transparency, as they function as “black boxes”, making it difficult for clinicians to understand and trust their predictions^10–12^. This has led to the emergence of explainable artificial intelligence (XAI) methods, which seek to enhance interpretability. In the medical field, these are of particular importance, as regulatory frameworks such as the EU AI Act^13^ mandate transparency and accountability. These led to the development and application of XAI frameworks, especially for ECG DL models, demonstrating that, to a certain degree, DL models learned features similar to cardiology textbook knowledge^14–16^.

Currently, alternative graph-based signal representations have been proposed, whereby biosignals are transformed into explicit graph structures prior to learning. Kultana and Türker^17^ have shown that converting ECG time series into graph representations, such as weighted visibility graphs, enables DL models to utilize the signal’s structure, leading to competitive performance. This demonstrates that clinically relevant information can be effectively captured in graph form. Aljanabi and Türker^18^ have employed coherence-based time-graph representations to model dynamic functional connectivity in electroencephalograms (EEGs) to detect Alzheimer’s disease.

With the rise in computational resources, graph neural networks (GNNs)^19^ gained attention due to their capacity to model complex data structures. Their application to various medical tasks, including disease prediction, drug discovery, and medical imaging analysis, has shown promising results^20–22^.

Graphs offer the advantage of modeling complex relationships and incorporating domain knowledge, making them particularly well-suited for multi-lead ECG signals, which represent differences in electric potentials and several challenges for their optimal representation: Since the 12-leads are derived from ten electrodes, they are not all linearly independent mathematically and there are eight independent and four redundant leads^18^; however, all 12 leads are clinically important. Each provides a unique anatomical view of the heart with different importance depending on the disease of interest and therefore, a priori removal of one of the leads is not feasible. Instead, several works were published making use of GNNs to represent 12-lead ECGs. For instance^23^, proposed a GNN representation that considered both temporal and spatial connections, with the latter capturing inter-lead relationships. A similar approach was chosen by Qiang et al.^24^, and Zhao et al.^25^. Guo et al.^26^ used a knowledge-guided graph representation for the prediction of the location of MI. In contrast, Kan et al.^27^ introduced a graph construct based on wavelet coefficients, focusing on frequency relationships rather than inter-lead spatial dependencies.

Despite these advances, the field remains in its infancy, and several challenges persist in applying GNNs to ECG processing. Best-practice guidelines for transforming 12-lead ECG data into graph structures remain undefined, mainly because systematic evaluation of whether GNN architectures adequately capture both spatial and temporal dependencies is lacking. Furthermore, none of the current state-of-the-art papers^23–25,28^ have published comprehensive source code, detailing the construction of the graph structure, which limits the reproducibility of results.

To address these unmet needs, we propose a complete, open-source pipeline for 12-lead ECG classification using GNNs, that enables insight into the GNNs’ decision-making process through explainability techniques. As a use case, the task of ECG classification is chosen, with a focus on MI localization, to quantify the extent to which the spatial connections of the GNNs are suitable.

## Results

### Classification results

**Fig. 2:**
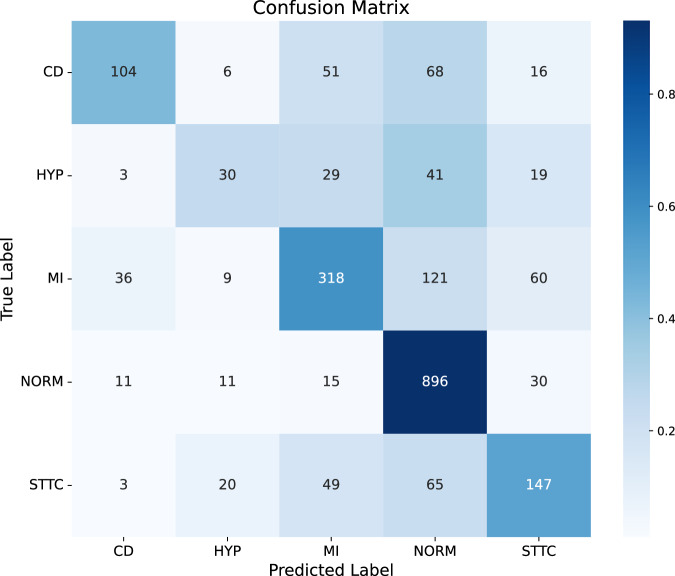
Confusion matrix for the diagnostic superclass classification (Task 1) on PTB-XL.

The proposed network was trained using the PTB-XL^29^ dataset, as described in section 4. The trained model performs two predictive tasks. Task 1 refers to the classification of ECG recordings into the five superclasses, and Task 2 refers to classifying MI subtypes using ECG recordings according to their localization within the heart. The classification performance for Task 1 is shown in Fig. 2.

The model demonstrates strong performance on the test set in recognizing normal ECG patterns (NORM, control samples), correctly classifying 899 out of 963 NORM samples, which aligns with its high recall of 0.93 (see Table 1).

**Table 1:** Class-specific results of precision, recall, and F1 score for the diagnostic superclass classification

Superclass	Samples	Precision	Recall	F1 score
CD	245	0.66	0.42	0.52
HYP	122	0.39	0.25	0.30
MI	544	0.69	0.58	0.63
NORM	963	0.75	0.93	0.83
STTC	284	0.54	0.52	0.53

However, notable misclassifications occur in other classes, particularly in MI and conduction disturbance (CD). A considerable number of samples of MI were incorrectly labeled as NORM (121 samples) or as ST/T Change (STTC) (60 samples), suggesting that these conditions share overlapping ECG features. Similarly, CDs were often confused with MI and NORM, with only 104 cases correctly classified. The Hypertrophy (HYP) class posed the most considerable challenge, as its samples were widely misclassified across multiple categories, reflected in its low recall of 0.25 and F1-score of 0.30. Notwithstanding these misclassifications, the model achieves an overall accuracy (ACC) of 0.69, a weighted F1-score of 0.68, a Matthews Correlation Coefficient (MCC) of 0.55, and a multiclass Area Under the Receiver Operating Characteristics Curve (AUC) of 0.86. Among the diagnostic categories, the best performance is observed for NORM (F1-score of 0.83), followed by MI (0.63) and STTC (0.53). However, CD and HYP exhibit lower F1-scores of 0.52 and 0.30, respectively, suggesting the necessity for enhanced class discrimination.

**Fig. 3:**
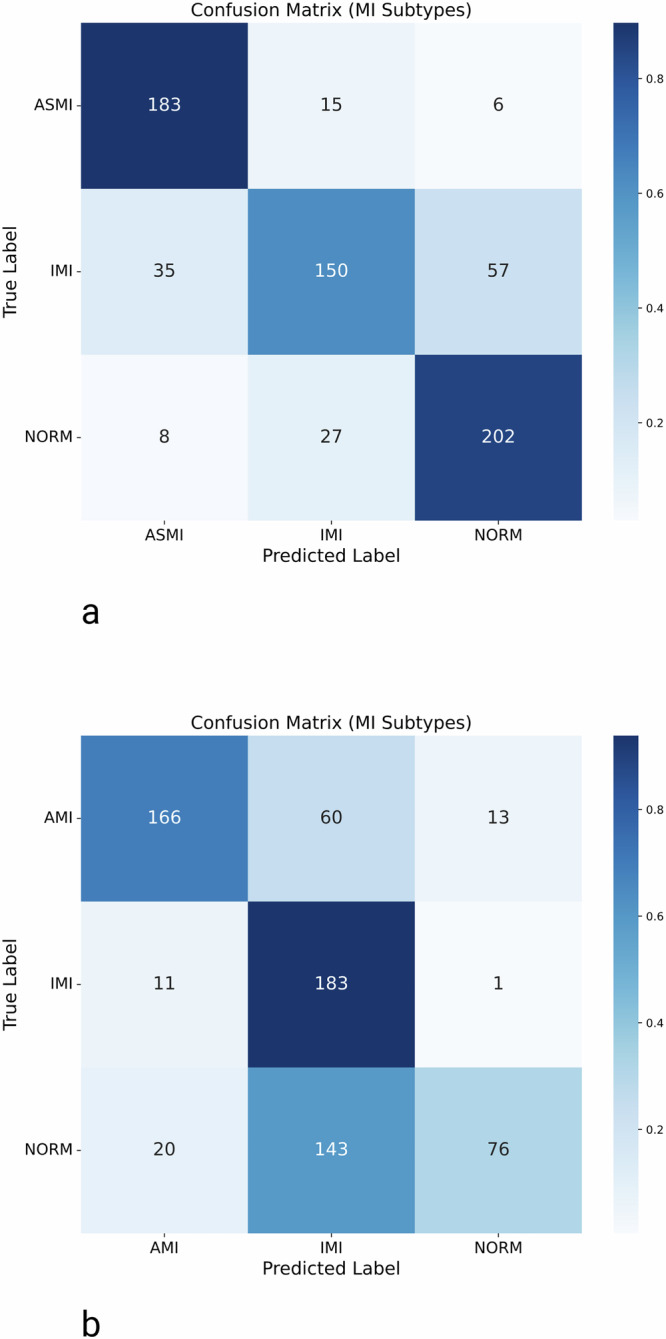
Confusion matrices illustrating the classification performance of the MI localization (Task 2) on the two datasets. **a** Shows the model’s performance in localizing MI on PTB-XL, while **b** depicts the results on the SHIP dataset.

While the model demonstrated good performance in the diagnostic superclass classification of Task 1, it achieved higher precision in the finer-grained MI subtype classification (Task 2) on PTB-XL. The classification results for the MI subclass are illustrated in Fig. 3a, with detailed performance metrics presented in Table 2. The network achieved an overall ACC of 0.78, a weighted F1-score of 0.78, an MCC of 0.68, and a multiclass AUC of 0.92, reflecting a moderate yet reliable ability to distinguish between MI subtypes.

**Table 2:** Class-specific results of precision, recall, and F1-score for MI localization on the PTB-XL dataset and the external validation dataset

Subclass	Samples	Precision	Recall	F1 score
ASMI PTB-XL	204	0.81	0.90	0.85
IMI PTB-XL	242	0.78	0.62	0.69
NORM PTB-XL	237	0.76	0.85	0.80
AMI SHIP	239	0.84	0.69	0.76
IMI SHIP	195	0.47	0.94	0.63
NORM SHIP	239	0.84	0.32	0.46

The model demonstrated a particularly good performance for the IMI and ASMI classes, with F1-scores of 0.85 and 0.69, respectively.

The generalizability of the trained model was evaluated on an external population-based dataset, with classification results shown in Fig. 3b and detailed performance metrics provided in Table 2. The model for Task 2 on the SHIP^30^ dataset achieved a multiclass AUC of 0.87, a weighted F1-score of 0.62, and an MCC of 0.51, indicating a moderate ability to generalize to unseen data. The model demonstrated a high level of recall for the IMI class (0.94), although this was accompanied by reduced precision (0.47), resulting in an F1-score of 0.63. Given the absence of more precise ASMI annotations in SHIP, our focus is on identifying AMI cases. The AMI class was also detected with high performance, achieving an F1-score of 0.76. In contrast, the classification performance for the NORM class was substantially lower, with a recall of only 0.32 and an F1-score of 0.46. This finding suggests that there are difficulties in identifying healthy controls within the external dataset. To investigate potential dataset shift, we compared QRS durations, a key ECG feature, between the cohorts using the Mann-Whitney-U test. Results showed significantly longer QRS durations in SHIP (*p*-value = 7.2 × 10^−13^ and cliff’s delta: 0.38) compared to the PTB-XL test set and significantly longer QRS durations in SHIP (*p*-value = 2.2 × 10^−^^19^ and cliff’s delta: 0.37) compared to the PTB-XL train set. All results can be found in Supplementary Fig. 1 and Supplementary Table 1.

### Explainability

**Fig. 4:**
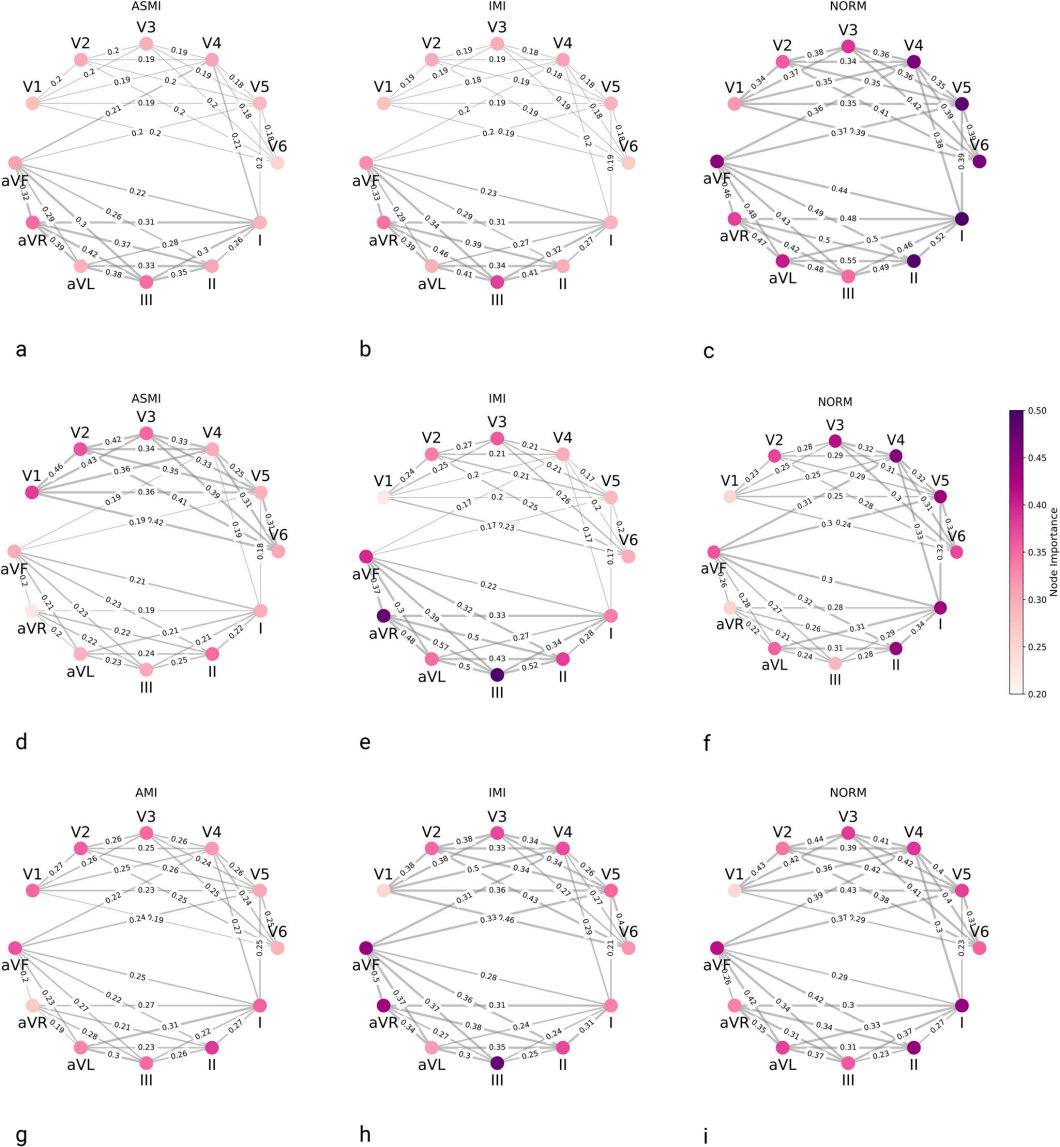
Average node and edge importance computed by GNNExplainer across samples for different classification tasks. Node importances are unitless and color-encoded from 0.2 to 0.5. Edge importances between nodes are written on the corresponding lines, with the line depth also encoding its importance. **a**–**c** Show the results for the MI subclasses obtained by the diagnostic superclass classification on the PTB-XL dataset: **a** ASMI, **b** IMI, and **c** control (NORM). **d**–**f** Depict MI localization on the PTB-XL dataset: **d** ASMI, **e** IMI, and **f** control (NORM). **g**–**i** Present MI localization on the SHIP dataset: **g** AMI, **h** IMI, and **i** control (NORM).

To investigate the explainability of the model, GNNExplainer^31^ was employed on true positive samples, since these cases reflect instances in which the model made correct predictions, making them suitable for interpreting the learned decision patterns. This results in 1495 samples for Task 1, 535 samples for Task 2 on PTB-XL, and 425 samples for Task 2 on the SHIP dataset. In order to provide a more comprehensive overview of the behavior of the trained GNN model, the average node and edge importance across these samples was visualized in Fig. 4.

For the classifier trained on Task 1, the superclass classification results can be found in Supplementary Fig. 2. The results for MI and CD, presented in Supplementary Fig. 2a, b, did not reveal any consistent or dominant patterns across ECG leads. In Supplementary Fig. 2c, the chest leads, especially V5 and V6, are highlighted, which are consistent with clinical practice, as the hypertrophy index, the Sokolow–Lyon index (SLI), is calculated using these leads^32^. The investigation revealed no specific region of the signal that exhibited recurrent importance, thereby suggesting that the model does not rely on a fixed set of leads for its predictions. This absence of a discernible trend can be ascribed to the heterogeneous nature of each superclass, which frequently encompasses multiple disease subtypes that manifest in disparate ECG regions. Furthermore, when visualizing the explanations per disease subtype using the same superclass-trained network, the largest class within each superclass was generally well represented. However, no distinct or recurrent patterns of important leads or connections were observed across the subtypes. This suggests that the model’s decision-making is distributed and not focused on specific ECG leads for finer-grained distinctions. To investigate the differences in the importance of leads across the two models of Task 1 and Task 2, explainability results are compared in Fig. 4. In particular, Fig. 4a–c shows the results for the myocard infarction localization using the network trained on the superclass classification task. In Fig. 4a, b, the model allocated the highest node importance scores to leads III and aVR. The connections between those leads also received the highest importance scores. These findings align with the focus that would be expected for IMI patients. Given the absence of clear patterns in the superclass classification setting, a more fine-grained analysis of the model trained for MI subtype classification is conducted.

The mean node and edge importance for Task 2 on PTB-XL were visualized across the MI subclasses, and the control class in Fig. 4d–f. In the context of the ASMI class, which can be seen in Fig. 4d, the model allocated the highest importance scores to the anterior precordial leads, with V1, V2, and V3 exhibiting node importances of more than 0.4. The connections among these leads, particularly V1–V2, V2–V3, and V3–V4, also received strong edge weights (up to 0.46), indicating a localized and coherent subgraph that aligns well with the known clinical relevance of these leads in detecting anterior-septal MI.

In the case of IMI (Fig. 4e), the importance distribution shifted towards the inferior leads. Leads III, II, and aVR demonstrated high node importance values, with III and aVR attaining more than 0.45. The model highlighted dense and high-weighted connections between these leads, particularly the edges between aVR–III, aVR–aVL, and II–III, where edge importances exceeded 0.5. This focus on the inferior and right-sided leads corresponds to standard diagnostic criteria for inferior infarction, thereby reinforcing the model’s physiological plausibility. A notable finding was the emergence of aVR as a prominent lead. Ruiz-Mateo et al.^33^ found that ST elevation in aVR is infrequent and not predictive in MI, but it has been reported as an independent predictor of cardiogenic shock. The model’s focus on aVR may thus reflect this clinical nuance, indicating its potential role in identifying patients at higher risk of severe complications.

In contrast, the control class, as shown in Fig. 4f, exhibited a more even distribution of node and edge importance across the ECG graph. No single lead dominated the importance map, particularly leads I, II, V3, V4, V5, and V6, receiving similarly high emphasis. The edge importance was similarly balanced, with no strong focal regions of attention. This diffuse representation is consistent with the absence of pathological patterns in healthy ECGs and provides a meaningful contrast to the concentrated lead importance observed in infarct classes. The comparison confirms that the model adapts its internal representations according to the presence or absence of disease, paying more selective attention to diagnostic regions in pathological cases, while maintaining a holistic view under normal conditions.

The mean node and edge importance scores on the SHIP dataset are visualized in Fig. 4g-i, and have a strong resemblance to those derived from the PTB-XL test set. For AMI (Fig. 4g), the precordial leads V1–V3 received higher levels of attention, both on the node and edge levels. In a similar manner, on the IMI (Fig. 4h) class, leads III, aVF, and aVR were given particular emphasis. The NORM class (Fig. 4i), however, showed higher importance scores on the limb leads than chest leads.

To assess time-related contributions, we quantified the importance of edges within each lead. In ASMI, temporal edge importance was highest in V1–V3 and remained elevated in V6. In IMI, the strongest temporal importance occurred in limb leads II and III with marked reciprocal patterns in aVR and aVL, while precordial leads showed lower values. NORM exhibited a comparatively flat distribution across leads, without localized temporal dominance. Detailed values are provided in Supplementary Table 2 and Supplementary Fig. 3.

## Discussion

The clinical deployment of artificial intelligence (AI) solutions for assessing CVD risk in 12-lead ECG is currently hindered by their limitations in interpretability and explainability. While recent studies demonstrate the potential of GNNs, widely adopted best-practice guidelines, standardized ECG graph construction procedures, and inherent explainability remain limited. To address these challenges, we present xGNN4MI, an open-source framework for GNN-based ECG modeling that emphasizes reproducibility and interpretability. The primary contribution of xGNN4MI lies in providing a transparent and configurable reference pipeline for transforming 12-lead ECG signals into graph representations, training GNN models, and interpreting their predictions. This will ultimately enable future studies to systematically evaluate alternative graph construction strategies. Specifically, our contributions are threefold: (i) we provide a framework that facilitates modeling clinically relevant spatial relationships between ECG leads and their temporal dynamics through an explicitly documented ECG graph construction procedure, as well as subsequent ECG-GNN training and evaluation. Standardized parameters and straightforward usage enable reproducibility and accessibility for future research. (ii) We integrate the existing GNNExplainer method in combination with task-specific cohort-level XAI evaluation and visualization routines to identify ECG leads and inter-lead connections that are most influential to the model’s decision-making process, enabling a systematic comparison of the results with established clinical knowledge and a thorough validation by clinical experts. This combination facilitates a more transparent understanding of which leads and inter-lead connections contributed to specific predictions. (iii) We evaluated xGNN4MI on two challenging, clinically relevant ECG classification tasks: (1) diagnostic superclass classification, and (2) localization of MI. Therefore, the ECG-GNN was trained on the open-source PTB-XL dataset and externally validated on the population-based cohort study SHIP. Our hyperparameter tuning focused on critical parameters (patch size, epochs) identified in prior work in ref. ^23^, though more exhaustive methods (e.g., random search) could be explored in future studies.

In the first task, the model demonstrated strong performance, achieving an AUC of 0.86, comparable to that reported by Zhang et al.^23^ with 0.88 and Zhao et al.^25^ with 0.91. However, the emphasis of this work was placed on providing a robust environment for other researchers that can be adapted to specific use cases, but the predictive performance was not optimized by excessive hyperparameter tuning. Lower recall and F1-scores were observed for CD and HYP, suggesting increased challenges for the network training in these classes. One possible contributing factor is the clinical heterogeneity associated with these conditions, which may complicate class separation under a single-label formulation. Consequently, improved performance for these classes may benefit from alternative approaches to feature disentanglement and additional input modalities towards multimodality. For the second task, the same model architecture and hyperparameters were used without further tuning to classify MI subtypes. This enabled an evaluation of the model’s generalizability across related diagnostic tasks. The selection of participants was conducted through a matching process, whereby subjects were categorized based on age group and sex, aligning them with the demographic parameters of IMI patients. This approach ensured a high degree of demographic comparability, facilitating effective analysis and interpretation of the data.

Our second main goal was to enhance interpretability by assessing model explainability using the GNNExplainer framework. For ASMI cases, node and edge importance were concentrated in the anterior leads (V1–V3), whereas IMI predictions emphasized the inferior leads (II, III, and aVF). This demonstrates the high agreement between learned GNN features and physiological knowledge. Notably, aVR was emphasized in IMI cases, which is consistent with recent clinical findings linking aVR to cardiogenic shock, suggesting that the model may have identified subtle yet clinically significant patterns^33^. In contrast, diffuse attention was exhibited across leads by the control group (NORM), consistent with an absence of pathology. These findings confirm that lead-specific representations were learned by the GNN, supporting the model’s pathophysiological plausibility. At the level of MI superclass classification, explainability patterns across MI subclasses were largely similar and did not exhibit clearly distinct lead-level relevance profiles.

Although the explainability patterns were consistent for Task 2 across the datasets, the model’s classification performance degraded notably for the NORM class on the SHIP dataset. This suggests that feature relevance alone does not guarantee robust generalization. While the model effectively localized infarction in AMI and IMI cases, it frequently misclassified healthy controls, as evidenced by a recall of 0.24. This trend is consistent with previous studies on GNNs for ECG classification^26^. The findings demonstrate a domain shift in the control group between the PTB-XL and SHIP datasets, presenting a statistically significant difference in QRS duration between the two cohorts. The attention given to leads and lead-pair interactions closely mirrors established electrocardiographic criteria for diagnosing MI subtypes, suggesting that the GNN has not only learned to classify correctly, but also to rely on physiologically meaningful features. Beyond the scope of retrospective interpretation, insights into explainability may inform future model adaptation. For instance, consistent lead and inter-lead relevance patterns, as observed between PTB-XL and SHIP, suggest that the model relies on stable, physiologically meaningful representations. Moreover, additional disease-focused physiological network structures, such as suggested^26^, may improve the performance of specific ECG classification tasks, which can be supported by the modular structure of the xGNN4MI framework.

While GNNExplainer offers valuable insights into the model’s decision-making process by identifying key nodes and edges, it has several limitations. First, the method assumes that the most influential subgraphs are structurally connected. However, in physiological signals such as ECGs, important relationships often exist between distant leads (e.g., limb and chest leads), which explainability methods that focus only on connected subgraphs may not capture. Second, although we additionally quantify the importance of edges linking consecutive temporal patches, GNNExplainer does not explicitly model temporal dynamics, which can be crucial in ECG data, where pathological patterns may appear only during specific time windows. Therefore, future research may explore these aspects via incorporating distant relationships of timely patterns, e.g., via PGMExplainer^34^ or time-aware attribution techniques^35^.

Additionally, one may address the model’s limitations in accurately identifying and classifying rare disease classes, and in reflecting the multi-label nature of clinical ECG interpretation. In the present study, each ECG was assigned a single dominant diagnostic label, although multiple diagnostic annotations may coexist for a given patient. While this simplification enables controlled evaluation and clearer interpretation, it does not fully capture real-world comorbidities. This objective should be pursued in future studies through two primary avenues: first, by expanding the scope to encompass multi-label classification; and second, by integrating supplementary explainability methods to provide a more comprehensive understanding of the model’s behavior. By advancing the interpretability and robustness of GNN-based ECG analysis, this research facilitates the development of trustworthy, clinically applicable AI-based systems for CVD diagnosis.

## Methods

**Fig. 5:**
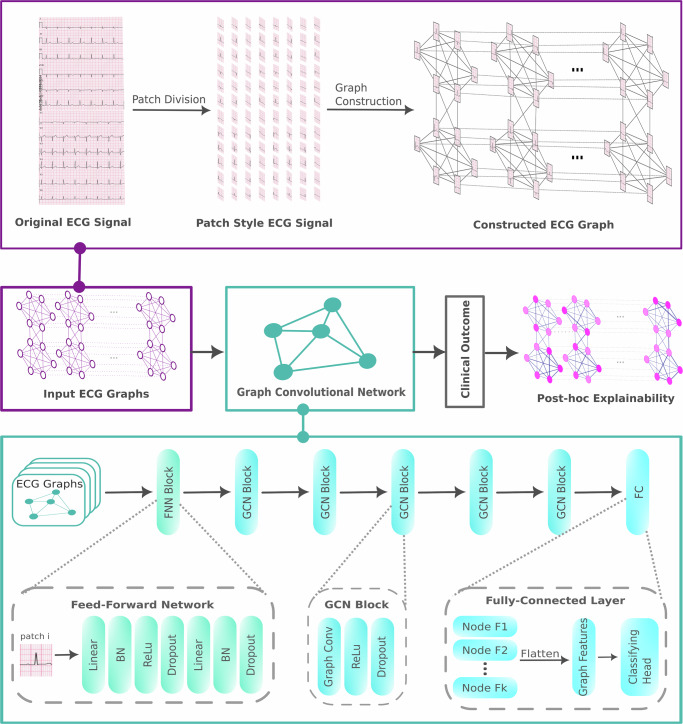
Schematic workflow: the nodes represent the signals, and the edges represent the physiological connections that can be created in accordance with the electrode position and vector space of the lead systems. The graph is processed by the GNN, which gives the clinical outcome. The post-hoc explainability method is used to calculate the node and edge importance for each graph. This workflow is displayed for all leads as ${\mathcal{S}}$ and the spatial lead connectivity in leads V4, V5, I, and aVF.

With the growing demand for interpretable machine learning models in clinical diagnostics, this study explores the potential of GNNs for ECG classification while integrating XAI techniques to enhance the transparency and trustworthiness of the model’s predictions. We suggest a methodological pipeline providing explanations of ECG classifications, as shown in Fig. 5. A GNN is trained on an ECG dataset to perform classification tasks. GNNExplainer is then used to analyze the learned graph structures and feature attributions, thereby assessing the GNN’s suitability for practical ECG interpretation. The pipeline is then evaluated using an external, population-based cohort study.

### Datasets

PTB-XL^29^ is a publicly available ECG dataset consisting of 21,799 clinical 12-lead ECGs from 18869 patients, each 10s in length. ECG signals are available in high resolution (500 Hz sampling rate) and low resolution (100 Hz sampling rate). To optimize computational efficiency while maintaining signal content, the high-resolution 500 Hz ECG signals were used and down-sampled to 250 Hz using polyphase resampling with anti-aliasing to balance diagnostic fidelity with efficiency and to align 400 ms patches to exactly 100 samples. A spot-check on the same splits at 100 Hz and 500 Hz (patch size *p* = 25) confirmed that 250 Hz preserves diagnostic content with modest differences relative to 500 Hz while reducing sequence length and computation. No further filtering or preprocessing was applied. To ensure reproducibility and prevent data leakage, the dataset partitioning outlined in ref. ^29^ was used, which follows the inter-patient paradigm, where ECG signals from the same patient are not present in the training, validation, and test sets simultaneously. Only ECGs that were marked as validated by human raters were incorporated, reducing the dataset to 15895 ECGs, to ensure a high-quality dataset for model training.

Apart from the raw signals, PTB-XL includes detailed metadata. Two levels of metadata were used, the five diagnostic superclasses: NORM, MI, STTC, CD, and HYP, as well as two subclasses of MI, namely IMI and ASMI. For the MI subclass classification task, we performed undersampling of the control cases (NORM) to address class imbalance. Patients from the NORM category were matched to IMI patients on sex and binned age group (<30, 30–45, 45–60, and >60), using the predefined partitioning from PTB-XL to ensure demographic comparability and prevent data leakage. Only cases validated by human raters were used, resulting in a total of 1341 NORM, 1372 IMI, and 1435 ASMI samples for the MI subclass classification task. The final per-class sample distribution used for the diagnostic superclass classification task was as follows: 7836 NORM, 3441 MI, 2093 STTC, 1699 CD, and 826 HYP samples. An overview of the sample distributions for both classification tasks and the validation data is provided in Table 3.

**Table 3:** Per-class sample distribution of the different tasks and datasets

	Task 1 PTB-XL	Task 2 PTB-XL	Task 2 SHIP
Per-class samples	7836 NORM	1341 NORM	239 NORM
	3441 MI	1372 IMI	195 IMI
	2093 STTC	1435 ASMI	239 AMI
	1699 CD		
	826 HYP		

Task 1 is the superclass classification task, and Task 2 is the MI localization task.

As an external validation dataset, ECG recordings from the Study of Health in Pomerania (SHIP)^30^ were utilized. SHIP is a large-scale, population-based cohort study conducted in northeastern Germany, in which extensive medical and sociodemographic data, including 12-lead ECGs, were collected from adult participants. For this study, data from SHIP-0 to SHIP-3, as well as SHIP-TREND-0 and SHIP-TREND-1, were used. This dataset is collectively referred to as SHIP in the following. All subjects underwent examinations in accordance with the SHIP protocol, which included 12-lead ECG acquisition of 10 s length. For the purposes of this study, cardiological data related to MI were used, specifically cases corresponding to AMI and IMI.

To ensure compatibility with the training data, all ECG signals were down-sampled to 250 Hz using polyphase filtering. No further filtering or preprocessing was applied. A total of 195 IMI cases and 239 AMI cases were extracted from the SHIP dataset. In this particular context, due to the lack of a more specific ASMI annotation in SHIP, we concentrate on the identification of AMI cases. The latter includes further subdivision into anteroseptal and extended anterior patterns, but are not always sharply delineated in routine clinical practice and can partially overlap in both presentation and interpretation. Additionally, 239 control cases (NORM) were randomly selected and matched to the IMI samples based on sex and binned age groups (<30, 30–45, 45–60, >60).

Ethical considerations. The SHIP study adhered to the recommendations of the 1964 Declaration of Helsinki. The medical ethics committee of the University of Greifswald approved the study protocol, and both oral and written informed consent were obtained from each study participant (approval number BB 39/08). Data for this work were acquired via the Transfer Unit for Data and Biomaterials of the Institute of Community Medicine at the University Medicine Greifswald.

### Graph construction and GNN model

In this section, we first introduce some basic notations and concepts, followed by a detailed description of the specific methods used in this study. While there are many potential tasks using GNNs on ECG data, in this work, we limit ourselves to the graph-level classification problem: A single 12-lead ECG is represented as an individual graph *G* and has one or more labels assigned from a set ${\mathcal{Y}}$. We denote a set of ECG graphs as ${\mathcal{G}}$, and the goal in classification is to train a GNN model ${f}_{\theta }:{\mathcal{G}}\to {\mathcal{Y}}$ with parameters *θ* that minimizes the function

$$\mathop{\min }\limits_{\theta }{\mathcal{L}}({\mathcal{G}})=\mathop{\sum }\limits_{{G}_{i}\in {{\mathcal{G}}}_{{\mathcal{T}}}}l({f}_{\theta }({G}_{i}),{Y}_{i}).$$

Here, *G*_*i*_ and *Y*_*i*_ denote the *i*-th graph from ${\mathcal{G}}$ and its ground truth label from ${\mathcal{Y}}$, respectively. The alignment between the model predicting *f*_*θ*_( ⋅ ) and the ground truth is measured using a use-case-specific loss function *l*( ⋅ , ⋅ ). After training *f*_*θ*_ on the training dataset ${{\mathcal{G}}}_{{\mathcal{T}}}$, its generalization capabilities are evaluated on an unseen test dataset.

**Fig. 6:**
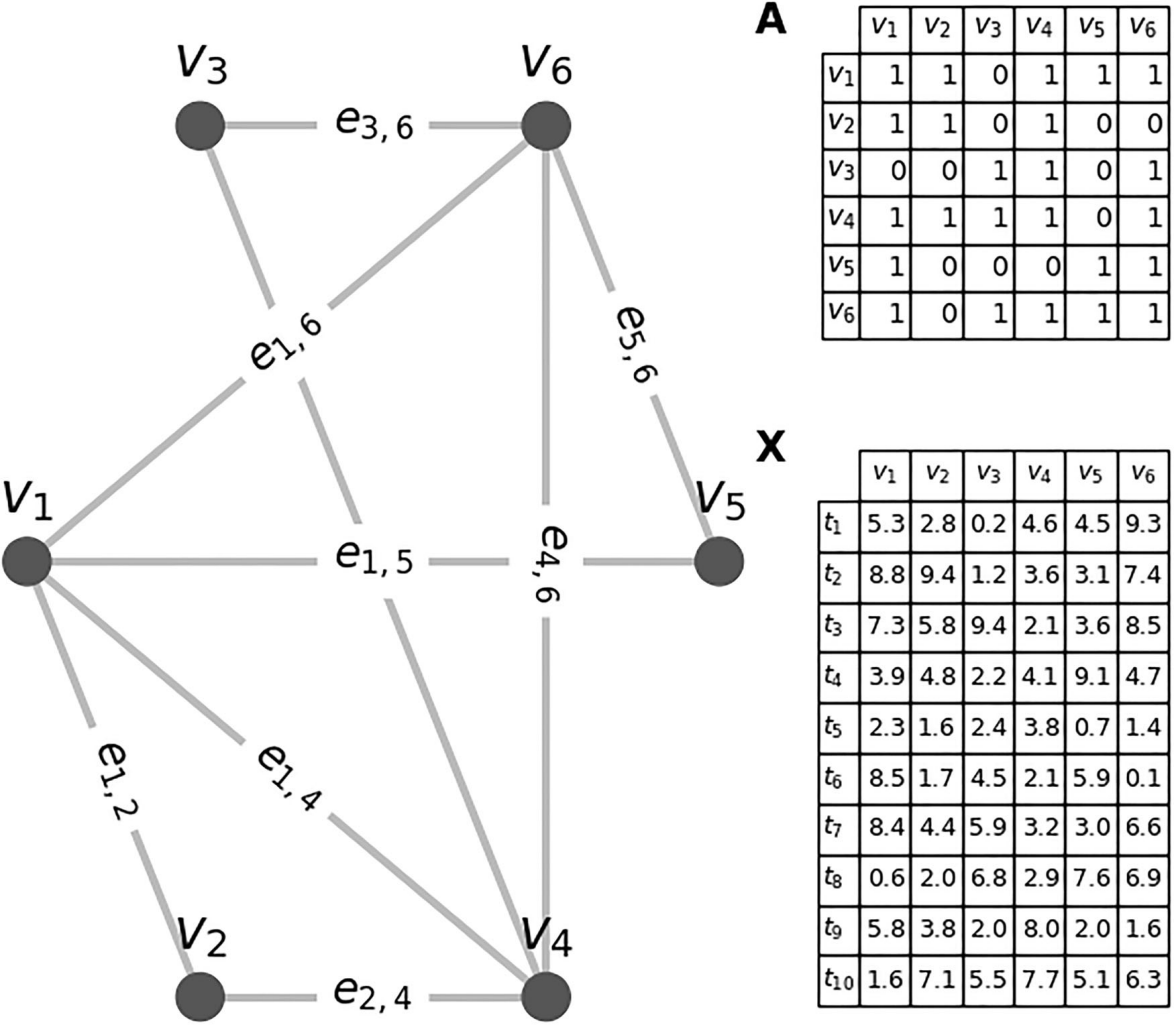
Exemplary graph *G* with six vertices and nine edges. For example, the vertices *v*_1_ and *v*_2_ are connected via the edge *e*_1,2_, thereby **A**_1,2_ = 1 in the adjacency matrix *A*. In this toy example, every feature vector **x** consists of a time series of *d* = 10 values and is stored in the node attribute matrix **X**^*N*×*d*^.

As 12-lead ECG data has both spatial (leads) and temporal (milliseconds) dimensions, both aspects are explicitly encoded in the graph representation. The first step is to represent the ECG signal as a graph *G* = (*V*, *E*), which consists of vertices (one node per considered lead) *v*_*i*_ ∈ *V*, where *i* ∈ {aVL, aVR, aVF, I, II, III, V1,…, V6} and edges ${e}_{i,j}={({v}_{i},{v}_{j})}_{i\ne j}\in E$ representing the spacial and timely connections. The latter represents the connections between nodes and is represented as a binary adjacency matrix $A\in {{\mathbb{R}}}^{N\times N}$ where each matrix element *A*_*i*,*j*_ is 1 if nodes *v*_*i*_ and *v*_*j*_ are adjacent (connected) and 0 otherwise. Any node *v* contains a feature vector $x\in {{\mathbb{R}}}^{d}$ consisting of *d* values. Hence, the node attribute matrix *X*^*N*×*d*^ contains all feature nodes of the graph *G*^20^. Figure 6 shows a toy example of an exemplary *G* with 6 vertices that contain each a feature vector of length 10.

Table 4 shows the parameters for graph construction for ECG. The first open parameter specifies which subset ${\mathcal{S}}$ of the full 12-lead set {aVL, aVR, aVF, I, II, III, V1,…, V6} to use as vertices. Every set from its power set ${\mathcal{P(S)}}$ could be used except for the empty set, e.g., only the limb leads {aVL, aVR, aVR, I, II, III} or only the chest leads {V1,…, V6}. The second open parameter defines the spatial connections of the selected vertices, i.e., which edges are defined within the adjacency matrix. In this evaluation, connections follow clinically established lead groupings: (1) Limb leads form a fully connected subgraph, (2) chest leads form a separate fully connected subgraph, and (3) key bridging links (I, aVF, V4, and V5) are added based on performance based on Zhang et al.^36^ and anatomical proximity (inferior—anterior reciprocity across limb leads, strong local coupling between contiguous lateral precordials).

**Table 4:** Parameters for graph construction

Description	Possible Values
Lead subset	All leads = aVL, aVR, aVF, I, II, III, V1, ⋯, V6 Limb leads = aVL, aVR, aVR, I, II, III Chest leads = V1, ⋯, V6
Spatial lead connectivity	Fully connected components: limb leads, chest leads, V4, V5, I, and aVF
Number of patches	*p* = 1, 10, 25, 50, 100

The last open parameter is related to how the chosen leads are connected over time. Previous research has shown that it is beneficial not to store the entire 10s ECG data in a single, spatial graph, but to use smaller parts of the ECG for the nodes and connect the leads in the same spatial pattern^23^. These parts of the ECGs are referred to as patches and are connected over time, as depicted in Fig. 5. Patches were created through uniform, non-overlapping division of the 10s ECG. Thereby, the last open parameter in Table 4 defines the number of patches, which is inversely associated with the time of a patch, e.g., a number of 10 patches results in a 1s patch duration while a number of 1 patches results in a 10s patch duration. This ultimately defines the number of vertices *v*_*i*,*t*_ ∈ *V*, where *i* ∈ {aVL, aVR, aVF, I, II, III, V1,…, V6} and *t* ∈ {1…*p*}

GNNs are a DL methodology that has been extended to non-Euclidean domains^23^. Unlike traditional machine learning models that operate on grid-structured data, such as images or sequences, GNNs enable learning representations from complex, irregular structures by leveraging the connectivity and relationships between nodes. The core operation in a GNN is message passing, where each node updates its representation by aggregating information from its neighbors. This paradigm is particularly advantageous for modeling physiological signals, such as ECGs, where spatial and temporal relationships play a critical role in diagnosis. At layer *l* + 1, the representation ${h}_{i}^{(l+1)}$ of node *i* is computed based on its previous state and the states of its neighboring nodes:

$${h}_{i}^{(l+1)}=\sigma \left(\mathop{\sum }\limits_{j\in N(i)}{W}^{(l)}{h}_{j}^{(l)}\right),$$

where *N*(*i*) denotes the set of neighbors of node *i*, *W*^(*l*)^ is a trainable weight matrix at layer *l*, and *σ* is a non-linear activation function such as ReLU. This iterative process allows each node’s representation to be influenced by progressively larger regions of the graph as the depth of the network increases.

A fundamental variant of GNNs is the Graph Convolutional Network (GCN)^37^, which normalizes the adjacency matrix to prevent scale distortions in the aggregation process. The layer-wise propagation rule in a GCN is formulated as:

$${H}^{(l+1)}=\sigma \left({\widetilde{D}}^{-\frac{1}{2}}\widetilde{A}{\widetilde{D}}^{-\frac{1}{2}}{H}^{(l)}{W}^{(l)}\right),$$

where *H*^(*l*)^ contains the node embeddings at layer *l*, $\widetilde{A}=A+{I}_{N}$ is the adjacency matrix, *I*_*N*_ is the identity matrix, and $\widetilde{{D}_{ii}}={\sum }_{j}\widetilde{{A}_{ij}}$^37^.

To incorporate the physiological relationships between the leads, all 12 leads are included, following the approach by Zhang et al.^23^. All six limb leads were fully connected, and all chest leads were fully connected. Both lead systems were connected by connecting leads I, aVF, V4, and V5, following the findings of Zhang et al.^23^, who evaluated different lead configurations. Regarding the segmentation of the ECG in time, the signal was divided into 25 patches, i.e., a patch duration of 400 ms, as suggested by Zhang et al.^23^ and supported by preliminary tests on the PTB-XL dataset for Task 1. Each ECG graph was assigned a single diagnostic label, corresponding to one of the predefined diagnostic superclasses.

A GCN proposed by Kipf and Welling^37^ was employed to classify ECG graphs following an architecture aligned with the Spatial-Temporal Residual Graph Convolutional Network (ST-ReGE)^23^. Prior to graph convolution, node features are transformed by a Feed-Forward Network (FFN), which improves representational capacity and reduces the effects of over-smoothing. The FFN comprises fully connected layers, batch normalization, and dropout regularization to ensure robust feature extraction before aggregation. After this transformation, the model processes the data through five GCN layers, with each layer refining the node representations by aggregating the features of neighboring nodes. Each of these layers incorporates ReLU activation and dropout to maintain generalizability and prevent overfitting. Consistent with Zhang et al.^23^, skip connections did not improve performance in our setting. In a comparison with the number of patches *p* = 25, the plain GCN without skip connections achieved comparable results, while DenseNet^38^ and ResNet^39^ variants had higher parameter counts Supplementary Table 3. The final node embeddings are then reshaped into a unified feature vector and passed through a fully connected classification layer to map the learned representations onto a diagnostic superclass. Finally, the proposed GCN architecture and pipeline were employed for both classification tasks: diagnostic superclass classification and MI subtype localization. Although the training targets differ between the two tasks, no structural or architectural modifications were introduced, ensuring consistency across the experiments.

The training and evaluation process was conducted in accordance with the preliminary findings of Zhang et al.^23^. Initial screening using 500 epochs and various numbers of patches *p* ∈ {1, 10, 25, 50, 100} showed, that *p* = 25 achieved the best performance based on ACC on the validation set. Therefore, hyperparameter tuning using *p* = 25 and *p* = 50 was conducted using the validation set to ascertain the optimal model parameters. The tuning process involved the testing of different combinations of learning rates (0.001 and 0.0001), batch sizes (1, 10, 32, 64, 128, and 250), and epoch sizes (100 and 150). ACC was used as the primary performance metric to select the best configuration. Once the optimal configuration of hyperparameters, as listed in Table 5, had been identified, the model underwent training on concatenated training and validation splits, which were used for the hyperparameter tuning. Then, this model was finally evaluated on a separate test set.

**Table 5:** Parameters of model training

Symbol	Description	Values
p	Number of patches	25
b	Batch size	32
lr	Learning rate	0.001
e	Epochs	150

The same model architecture and hyperparameters determined through tuning on the diagnostic superclass classification task were subsequently applied to the MI subclass classification task without further tuning. This allowed for an assessment of the generalizability of the network configuration across related but more general diagnostic tasks.

For the final evaluation, the test set was utilized, and performance was assessed using multiple evaluation metrics, including ACC, weighted F1-score (*F*1), multiclass AUC with one vs rest, MCC, precision (*Pre*), and recall (*Re*):

$$\begin{array}{rcl}Acc & = & \frac{TP+TN}{TP+TN+FP+FN},\\ Pre & = & \frac{TP}{TP+FP},\\ Re & = & \frac{TP}{TP+FN},\\ F1 & = & \mathop{\sum }\limits_{i}^{C}{\beta }_{i}\left(2\frac{Pr{e}_{i}\times R{e}_{i}}{Pr{e}_{i}+R{e}_{i}}\right),\\ MCC & = & \frac{TP\times TN-FP\times FN}{\sqrt{(TP+FP)(TP+FN)(TN+FP)(TN+FN)}},\end{array}$$

where *TP* denotes true positives, *TN* true negatives, *FP* false positives and *FN* false negatives. The number of classes is denoted as *C*, and the proportion of observations in class *i* is *β*_*i*_.

### Explainability

Several taxonomies of XAI have been proposed for GNNs. For example, Yuan et al.^40^ provided a structured categorization of GNN explainability techniques, distinguishing between instance-level explanations, which focus on identifying influential nodes, edges, or subgraphs relevant to a specific prediction, also called local explanations, and model-level explanations, which aim to provide global insights into the decision-making process of the entire model. Furthermore, a range of methods can be employed to extract local and global explanations: instance-level methods encompass a variety of approaches. Gradient-based techniques use backpropagation to analyze the influence of input features. In contrast, perturbation-based methods evaluate the impact of selectively removing graph components by observing changes in the model’s output. Decomposition-based approaches are utilized to trace the flow of information through the model, with the objective of identifying critical paths or components. Finally, surrogate-based methods approximate complex models by employing simpler, more interpretable alternatives, thereby enhancing the transparency of the decision-making process. Building upon this work, Longo et al.^41^ conducted a comparative analysis of various explainability techniques for GNNs, often referred to as explainers, highlighting the relationship between different model architectures and their corresponding explainability performance. The study highlights several key challenges in GNN explainability, including the lack of standardized evaluation benchmarks, similar to those established for other data types^42^, and the difficulty of interpreting explanations in a clinically meaningful manner. The findings suggest that the effectiveness of different explainers depends on the underlying GNN architecture and the characteristics of the dataset, underlining the importance of domain-specific optimization strategies. These results are consistent with those from studies of other DL architectures^10,43,44^, which have shown that different XAI techniques can produce different relevance attributions for the same model and dataset, subsequently leading to inconsistencies in explainability outcomes.

Among the various instance-level explainability techniques, GNNExplainer has emerged as a widely adopted, model-agnostic approach for interpreting GNN predictions. Introduced by Ying et al.^31^, this perturbation-based method aims to identify the most important substructures – nodes, edges, and features—in a graph that contribute to a specific prediction. A notable advantage of GNNExplainer is its architecture-independent design, which facilitates the post-hoc generation of explanations without necessitating the retraining of the model.

Given a trained model *f*, an input graph *G* = (*V*, *E*), and associated node features *X*, GNNExplainer aims to find a compact subgraph *G*_*S*_ ⊆ *G* and a subset of node features *X*_*S*_ ⊆ *X* that are most relevant for a given classification decision. Let *f*(*G*) = *Y* represent the predicted class probability of the GNN model for the graph *G*. The GNNExplainer seeks to learn two soft masks *M*_*E*_ over the adjacency matrix *A* and *M*_*X*_ over the node features *X*. These masks indicate the most important edges and features, respectively, and are applied as follows:

$$\begin{array}{rcl}{A}^{{\prime} } & = & A\odot {M}_{E}\\ {X}^{{\prime} } & = & X\odot {M}_{X},\end{array}$$

where ⊙ denotes element-wise multiplication. The optimization objective is to maximize the mutual information *MInfo* between the prediction of the original graph *Y* and the prediction of the masked graph and features:

$$\max {G}_{S}\,MInfo(Y,({G}_{S},{X}_{S}))=H(Y)-H(Y| G={G}_{S},X={X}_{S}),$$

where *H*(*Y*∣*G* = *G*_*S*_, *X* = *X*_*S*_) is the conditional entropy given the selected subgraph and features, and *H*(*Y*) is the entropy of the prediction. The explanation masks are optimized using a gradient-based procedure. Starting from random initialization, the method performs forward passes through the original GNN using the masked inputs, computes the *MInfo*-based loss, and updates the masks through backpropagation. This process is typically repeated until convergence, typically within 200 iterations, after which the most relevant subgraph and features for the prediction can be extracted. Consequently, GNNExplainer is adopted in this study to gain insight into the decision-making process of the GNN models applied to ECG classification.

Here, the PYTORCH GEOMETRIC^45^ Python library was used to utilize the GNNExplainer. The GNNExplainer module was implemented after training the model, using individual ECG graph instances to provide localized, instance-specific verification of model predictions. In contrast to methods that necessitate alterations to the model or training procedure, GNNExplainer functions independently of the GNN architecture. The obtained masks highlight the most relevant nodes and edges that contribute to the model’s output. By using GNNExplainer, the graph components that drive classification outcomes can be highlighted, thereby improving transparency and enabling domain experts to better understand and trust model decisions. Explanation scores obtained from GNNExplainer are processed and visualized primarily at the cohort level by aggregating importance scores across patients within each diagnostic class, yielding stable ECG lead relevance patterns suitable for comparison with established clinical knowledge. While the visualization framework has been developed to support cohort-level analysis, it can be adapted to patient-level analysis by averaging importance scores across temporal connections within each lead. The fundamental GNNExplainer optimization has not been modified, all adaptations are implemented during the aggregation and visualization stage.

## Supplementary information

Supplementary information

## Data Availability

The data of the SHIP study cannot be made publicly available due to the informed consent of the study participants, but it can be accessed through a data application form available at https://fvcm.med.uni-greifswald.de/ for researchers who meet the criteria for access to confidential data.

## Code availability

The data analysis code will be freely available on GitHub following publication of the paper (https://github.com/HauschildLab/xGNN4MI). Please contact Miriam Cindy Maurer at miriamcindy.maurer@med.uni-goettingen.de.
