# Supplementary material for: xGNN4MI: explainability of graph neural networks in 12-lead electrocardiography for cardiovascular disease classification
Source: NPJ Digit Med. 2026 Feb 6;9:256. doi: 10.1038/s41746-026-02367-1 (PMC13021949; doi:10.1038/s41746-026-02367-1)
Supplement: Supplementary file 1 — Supplementary information [file 41746_2026_2367_MOESM1_ESM.pdf]

Supplementary Information

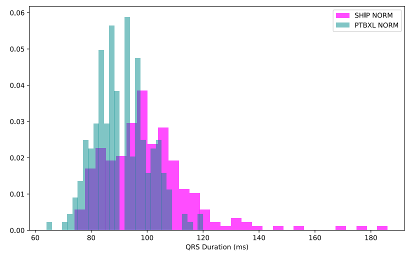

(a) QRS duration of the SHIP NORM patients and the test NORM patients of PTB-XL

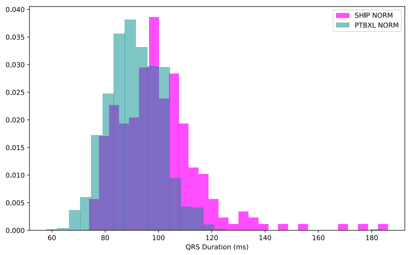

(b) QRS duration of the SHIP NORM patients and the train NORM patients of PTB-XL

Supplementary Figure 1: QRS duration comparison of healthy control patients (NORM) in PTB-XL and SHIP.

Dataset	U statistics	Mann-Whitney-U p-value	Cliff's delta
SHIP vs PTB-XL test	38616.0	$7.23 * 10^{-13}$	0.38
SHIP vs PTB-XL train	178764.5	$2.23 * 10^{-19}$	0.37

Supplementary Table 1: Statistical comparison of QRS duration of SHIP NORM patients with NORM patients of the train and test set of PTB-XL using Mann-Whitney-U test and Cliff's delta.

Disease	Summed temporal edge importance per lead											
	V1	V2	V3	V4	V5	V6	I	II	III	aVR	aVL	aVF
ASMI	23.99	23.12	22.01	15.96	16.12	20.69	8.79	11.71	11.56	9.92	10.97	9.19
IMI	11.62	12.42	12.71	8.48	8.39	11.59	11.38	22.38	26.33	26.03	21.29	11.20
NORM	11.98	12.57	13.59	13.79	13.17	13.56	14.13	15.18	12.98	11.62	13.67	13.75

Supplementary Table 2: Summed temporal edge importance per disease and per lead for the myocardial infarct localization classification on PTB-XL.

Architecture	batch size	lr	Accuracy	F1-score	F1-macro	MCC	AUC	Trainable Parameters
plain	32	0.001	0.696	0.678	0.572	0.562	0.878	96 749
resnet	10	0.001	0.701	0.676	0.545	0.562	0.893	100 757
densenet	128	0.001	0.704	0.682	0.552	0.567	0.884	104 209

Supplementary Table 3: Best configurations for different skip connections, testing batch size, and learning rate. All models have 5 GCN blocks, and the number of patches is equal to 25.

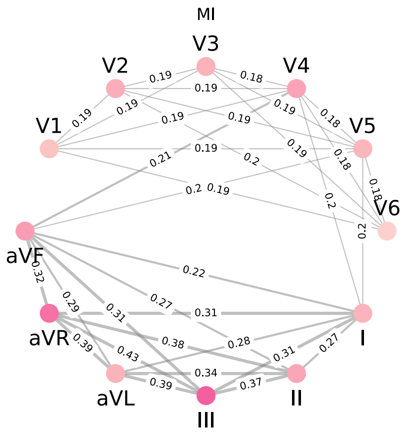

(a) Task 1 MI on PTB-XL

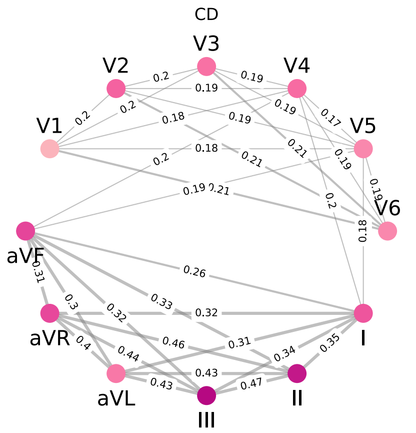

(b) Task 1 CD on PTB-XL

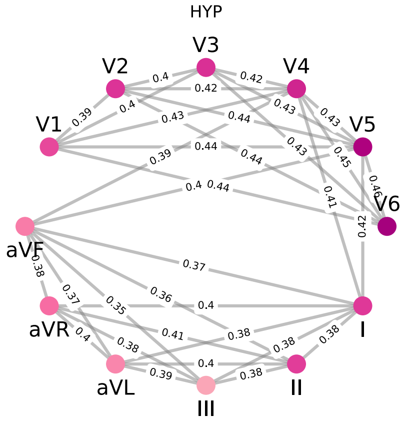

(c) Task 1 HYP on PTB-XL

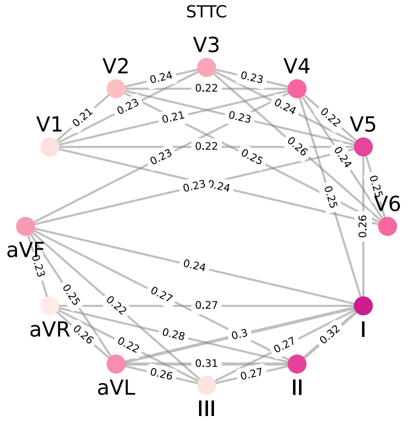

(d) Task 1 STTC on PTB-XL

Supplementary Figure 2: Average node and edge importance computed by GNNExplainer across samples for Task1. Node importances are unitless and color-encoded from 0.2 to 0.5. Edge importances between nodes are written on the corresponding lines, with the line depth also encoding its importance. Subfigures (a) to (d) show the results for diagnostic superclass classification: (a) myocardial infarction (MI), (b) conduction disturbance (CD), (c) hypertrophy (HYP), and ST/T change (STTC).

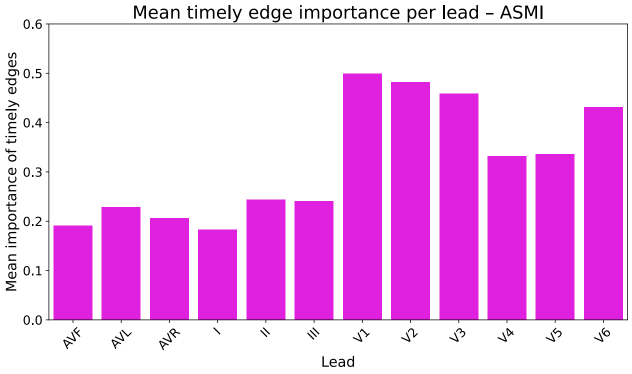

(a) Mean temporal importance per lead in ASMI

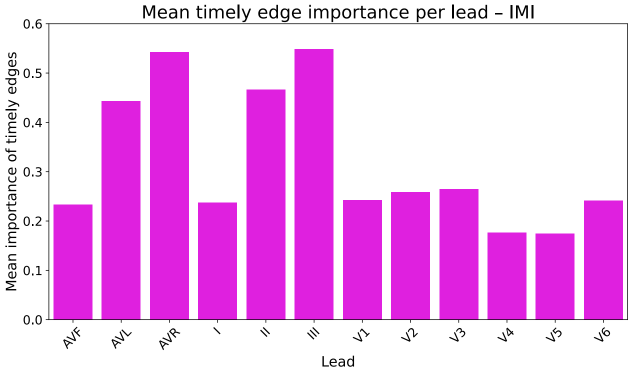

(b) Mean temporal importance per lead in IMI

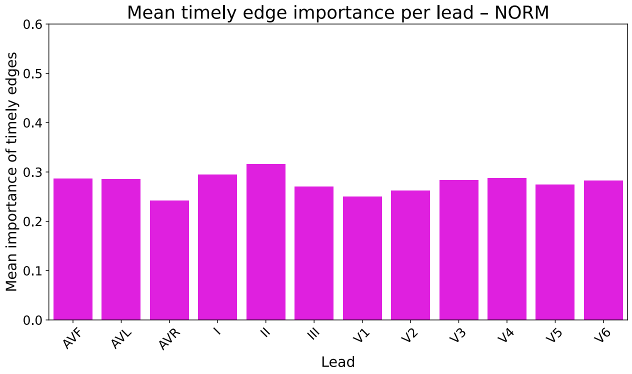

(c) Mean temporal importance per lead in NORM

Supplementary Figure 3: Distribution for mean timely edge importance per lead.
